# Clinical Importance of the Human Umbilical Artery Potassium Channels

**DOI:** 10.3390/cells9091956

**Published:** 2020-08-25

**Authors:** Margarida Lorigo, Nelson Oliveira, Elisa Cairrao

**Affiliations:** 1 CICS-UBI, Health Sciences Research Centre, University of Beira Interior, 6200-506 Covilhã, Portugal; margarida.lorigo@gmail.com; 2 FCS-UBI, Faculty of Health Sciences, Department of Medical Sciences, University of Beira Interior, 6200-506 Covilhã, Portugal; 3 UDI-IPG, Research Unit for Inland Development, Department of Social Sciences and Communication, Polytechnic Institute of Guarda, 6300-654 Guarda, Portugal; nelsonoliveira@ipg.pt

**Keywords:** potassium channels, vascular smooth muscle cells, human umbilical artery, vascular diseases, gestational hypertension, preeclampsia

## Abstract

Potassium (K^+^) channels are usually predominant in the membranes of vascular smooth muscle cells (SMCs). These channels play an important role in regulating the membrane potential and vessel contractility—a role that depends on the vascular bed. Thus, the activity of K^+^ channels represents one of the main mechanisms regulating the vascular tone in physiological and pathophysiological conditions. Briefly, the activation of K^+^ channels in SMC leads to hyperpolarization and vasorelaxation, while its inhibition induces depolarization and consequent vascular contraction. Currently, there are four different types of K^+^ channels described in SMCs: voltage-dependent K^+^ (K_V_) channels, calcium-activated K^+^ (K_Ca_) channels, inward rectifier K^+^ (Kir) channels, and 2-pore domain K^+^ (K_2P_) channels. Due to the fundamental role of K^+^ channels in excitable cells, these channels are promising therapeutic targets in clinical practice. Therefore, this review discusses the basic properties of the various types of K^+^ channels, including structure, cellular mechanisms that regulate their activity, and new advances in the development of activators and blockers of these channels. The vascular functions of these channels will be discussed with a focus on vascular SMCs of the human umbilical artery. Then, the clinical importance of K^+^ channels in the treatment and prevention of cardiovascular diseases during pregnancy, such as gestational hypertension and preeclampsia, will be explored.

## 1. Introduction

Potassium (K^+^) channels are usually predominant in the membranes of vascular smooth muscle cells (SMCs). These channels play an important role in regulating the resting membrane potential (MP) and vessel contractility—a role that depends on the vascular bed. Therefore, the physiological MP range in SMCs is essential to understand the K^+^ channel’s role in smooth muscle (SM) [1]. Overall, vascular SMCs have an MP ranging from −40 and −60 mV, depending on the type of blood vessel [2,3]. Thus, since MP is considerably more positive than the K^+^ equilibrium potential (E_K_; about −85 mV), it appears that the permeability of K^+^ does not completely dominate MP conductance. When E_K_ is substantially more negative than MP, the opening of K^+^-channels induces the K^+^ efflux into the extracellular medium, which can cause hyperpolarization or repolarization of the cell membrane, closure of the voltage-gated Ca^2+^-channels (VGCCs), decrease in calcium (Ca^2+^)-entry into the cell, and vasodilatation. On the contrary, the closing of K^+^-channels can cause depolarization, opening of VGCCs, increase of intracellular Ca^2+^, and vasoconstriction [1,2,4,5,6,7,8].

**Figure 1:**
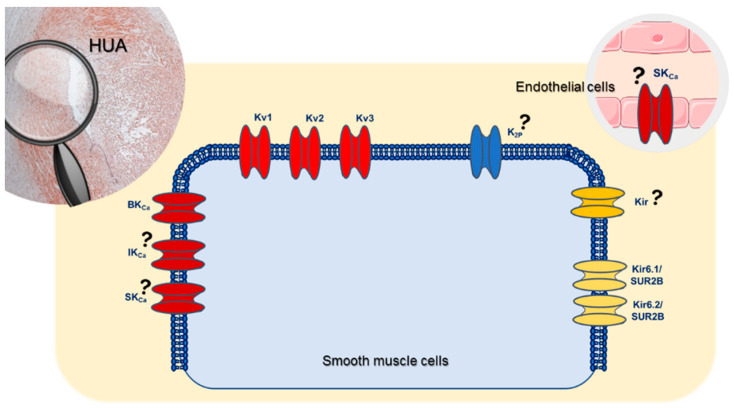
Schematic representation of the K^+^ channels present in human umbilical artery.

In the vascular SMCs, the presence of the various K^+^-channels varies and there is no homogeneous distribution. For example, there are 100–500 ATP-sensitive K^+^ (K_ATP_) and inward rectifier K^+^ (Kir) channels per cell, while voltage-dependent K^+^ (K_V_) and Ca^2+^-activated K^+^ (K_Ca_) channels are around 1000 to 10,000 per cell. Moreover, the electrophysiological measurements using the patch-clamp technique showed that the amplitudes of the basal potassium currents in each channel are very different [1,5]. Currently, there are four different types of K^+^ channels described in SMCs from the human umbilical artery: K_V_, K_Ca_, Kir, and 2-pore domain K^+^ (K_2P_) channels (Figure 1) [9,10]. However, the presence of intermediate-conductance Ca^2+^-activated K^+^ channels (IK_Ca_), one subtype of K_Ca_, has never been demonstrated [9,10].

The human umbilical artery (HUA) is a unique vessel that transports deoxygenated blood. This artery does not have nerve endings, so the regulation of its tonus is completely dependent on the locally released vasoactive mediators, substances carried by the bloodstream, and some ions, such as K^+^ and Ca^2+^ [11,12]. Therefore, HUA is much more susceptible to hemodynamic changes caused by vascular disorders [13]. Moreover, its SMCs appear to play an important role in the control of fetoplacental blood flow. Moreover, vascular SMCs are responsible for the vascular tone by responding to various hormonal and hemodynamic stimuli [9,14]. In this sense, being a fetal structure, the umbilical cord (UC) can be used as a window to learn about maternal dysfunctions and their impacts on fetal well-being [13], namely, regarding HUA K^+^-channels. The elucidation of the exact mechanisms regulating HUA contractile status is extremely important to detect potential targets for the treatment of pregnancy-related pathologies, mainly gestational hypertension and preeclampsia. 

Therefore, this review discusses the basic properties of the various types of K^+^-channels, including the structure and cellular mechanisms that regulate their activity and new advances in the development of activators and blockers of these channels. The vascular functions of these channels will be discussed with a focus on vascular human umbilical artery smooth muscle cells (HUASMCs). Then, the clinical importance of K^+^-channels in the treatment and prevention of cardiovascular diseases (CVDs) during pregnancy, such as gestational hypertension and preeclampsia, will be explored. 

## 2. The Importance of the K^+^ Channels in Physiological Regulation of HUA

Ion flux is one of the pathways responsible for regulating the arterial SM tonus. In this process, the activation of K^+^-channels plays a crucial role because their activation is mainly responsible for SM relaxation [7,15]. The K^+^ channels are the most abundant in SMCs and play a crucial role in the regulation of MP because they directly control K^+^ concentrations and indirectly control Ca^2+^ concentrations [1]. More specifically, the activation of these channels in vascular SMCs hyperpolarizes the cell membrane, inducing the closure of VGCC and thus decreasing the intracellular Ca^2+^ concentration ([Ca^2+^]_i_), giving rise to a vasorelaxation [2,4,16]. In addition, this channel activation can be originated through some vasoactive substances (e.g., nitric oxide (NO), natriuretic peptides (NP), and prostacyclins), which can activate kinase proteins [17]. 

Focusing on the HUA K^+^ channels, the presence of K_V_, large-conductance Ca^2+^-activated K^+^ channels (BK_Ca_), small-conductance Ca^2+^-activated K^+^ (SK_Ca_), and Kir, K_ATP_, and K_2P_ channels was demonstrated. On the other hand, the presence of IK_Ca_ channels has never been reported [15]. In addition, in HUA, the activation of K^+^-channels is one of the main mechanisms involved in its vasorelaxation. The K_v_ channels regulate MP in response to membrane depolarization, while BK_Ca_ channels respond to changes in intracellular Ca^2+^ levels [15,18,19,20,21]. The activity of the several types of K^+^-channels can be altered by several physiological factors, including intracellular Ca^2+^, cyclic nucleotides, and various signal transduction mechanisms. In HUA, the cyclic guanosine monophosphate (cGMP)-induced vasodilatation is mediated by K^+^-channels activation [9], and this effect is lower when HUA is contracted by potassium chloride (60 mmol/L) or serotonin [22]. Additionally, Cairrao et al. (2010) demonstrated that K^+^ currents (I_K_) are stimulated by NP, and this effect corresponds to the activation of BK_Ca_ and Kv channels through an increase of cGMP and protein kinase G (PKG) activation [23]. The same authors also observed that K^+^ currents (I_K_) are not stimulated by NO donors. In this sense, they concluded that the activation of BK_Ca_ and Kv channels is dependent on differences in the spatiotemporal distribution of intracellular cGMP. These results suggest that when a cyclase is activated, besides its hydrosolubility, intracellular cGMP is not uniformly distributed within the cell, and it is probably clustered in specific sites. Moreover, the authors found that phosphodiesterases (PDE; e.g., PDE3 and PDE5) are involved in this compartmentalization, regulating the pools of particulate and cytosolic cGMP. These results are suggestive that the particulate pool but not the soluble one, seems to be controlled by PDE5, while PDE3 appears to control the soluble cGMP pool exclusively [24,25]. Thus, these results are extremely important for the development of new drugs, since we can control the effect on the ion channels not only with drugs that act in K^+^ channels, but also with drugs that act in PKG and PDE.

In summary, further studies on the functional role and the expression of K^+^-channels in HUA are needed to clarify the mechanisms involved in relaxation. The expression and functionality of these channels may change in vascular- or pregnancy-related pathologies. Therefore, further studies are needed to understand how K^+^-channels can be an important pharmacological target in the development of new treatments, mainly in pregnancy hypertensive disorders. 

## 3. Diversity of K^+^ Channels in HUA 

### 3.1. Voltage-Dependent K^+^ (Kv) Channels

The voltage-dependent K^+^ channels, or Kv channels, are channels present in SMCs that are activated by depolarization. When a depolarization of SMCs occurs, even if small, there is an activation of L-type VGCCs, which leads to an increase in the Ca^2+^-influx and stimulation of the contractile apparatus. However, the influx of Ca^2+^ through L-type VGCCs associated with membrane depolarization can also inhibit Kv channels. There is the reason for why the activity of the Kv channels is important in regulating the excitability of SMCs and maintaining basal tone [2,7,20,26].

At the structural level, the Kv channels consist of four α-subunits, each containing six transmembrane α-helical segments, S1–S6, and a membrane-reentering P-loop (P) [27]. This ion-conduction pore is lined by four S5-P-S6 sequences, while the four S1–S4 segments, each containing positively charged amino acid residues (arginine and lysine) in membrane-spanning domain S4, detect changes in membrane potential and work as the voltage sensor [27,28,29]. Accessory subunits interact with the pore-forming α-subunits and modulate channel function and interactions with scaffolding and other proteins in macromolecular signaling complexes [2]. Kv channels are a family of 40 proteins classified into 12 subtypes (KV1-12) that can assemble as homo- or heterotetramers. This diversity generates a vast array of Kv current phenotypes that can differ in their kinetics, amplitude, and responses to different modulators. Tissue- and species-specific expression of Kv channel isoforms have also been described, which further contributes to Kv current heterogeneity [30]. The presence of several families of Kv channels has been demonstrated in vascular SMCs, namely, KV1, KV2, KV3, KV4, KV6, KV7, KV9, and KV11, with KV1 (KV1.1, KV1.2, KV1.3, KV1.5, KV1.6), -2 (K_V_2.1), and -7 (K_V_ 7.1–7.5) being particularly important (see review in [2]). Moreover, the surface abundance of Kv channels in arterial SMCs is regulated by several mechanisms (see review in [30]).

At the pharmacological level, Jackson et al. (2018) [2] and Dogan et al. (2019) [1]) have recently extensively reviewed the pharmacology of Kv channels in vascular SMCs. Briefly, the main Kv blockers are 4-aminopyridine (4-AP), tetraethylammonium (TEA), barium ion (Ba^2+^), glibenclamide, margatoxin, charybdotoxin, diphenylphosphine oxide-1 linopirdine, XE991, and HMR1556, of which the most widely used is 4-AP. Regarding the activators/agonists, there are only a few, and they are specific to Kv7 and Kv11 (please see the review in [2]). 

**Figure 2:**
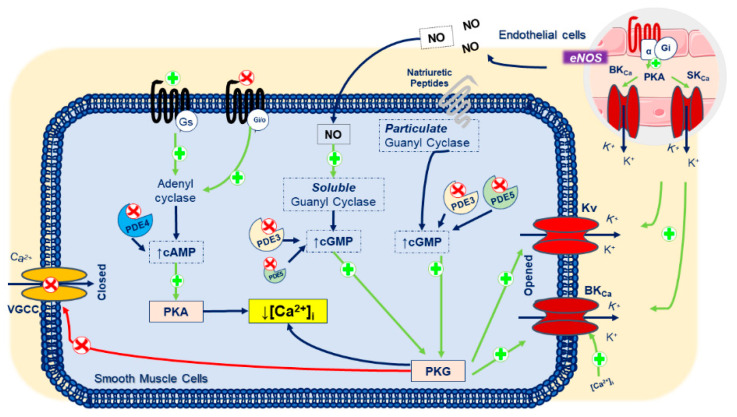
Schematic representation of the mechanisms implicated in activation of Kv and BK_Ca_ channels in human umbilical artery smooth muscle cells (HUASMCs). **LEGEND:** 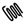
—G-protein coupled receptor; 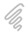
—peptide receptor; 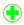/green arrows—stimulation; 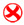/red arrows—inhibition; **?**—unknown mechanism; [Ca^2+^]_I_—intracellular Ca^2+^ concentration; BK_Ca_—large-conductance Ca^2+^-activated K^+^ channels; Ca^2+^—calcium; cAMP—cyclic adenosine monophosphate; cGMP—cyclic guanosine monophosphate; eNOS—endothelial nitric oxide synthase; K^+^—potassium; K_v_—voltage-gated K^+^ channels; NO—nitric oxide; PDE—phosphodiesterase; PKA—protein kinase A; PKG—protein kinase G; SK_Ca_—small-conductance Ca^2+^-activated K^+^ channels; VGCC—voltage-gated Ca^2+^ channels.

Regarding the Kv functional presence in HUA, it seems that these channels are not involved in determining basal contractile since the Kv blocker 4-AP had no effect on basal tonus of the HUA rings [18,21]. However, the relaxations induced by NO [31], testosterone [21], or levosimendan [32] are partially inhibited by 4-AP in HUA rings contracted with thromboxane mimetic U46619, serotonin, histamine, and KCl 60 mmol/L. Furthermore, Milesi et al. (2003), by patch-clamp recordings, demonstrated that I_K_ was partially inhibited by 4-AP [18]. Using a depolarizing voltage step protocol, the authors determined the presence of members of the Kv family sensitive to 4-AP [18]. Accordingly, our research group reported similar results in primary cultured SMCs obtained from HUA. I_K_ is stimulated by atrial natriuretic peptide (ANP), and this effect corresponds to the activation of BK_Ca_ and Kv channels through an increase of cGMP and PKG activation [23] (Figure 2). Recently, Martin et al. (2014) demonstrated the existence of members of Kv subfamilies in HUASMCs, namely, K_V_1 (except K_V_1.4, which presents inactivation), K_V_2, or K_V_3 [15]. However, the authors highlighted the importance of more studies with specific blockers to identify the different types of Kv channels. 

In summary, the Kv channels are the main determinants of vascular tone, and their presence in SMCs and HUASMCs is demonstrated. However, some differences at the family level were found. Members of the Kv subfamilies (Kv1, Kv2, Kv3, Kv4, Kv6, Kv7, Kv9, and Kv11) were found in SMCs, while in HUASMCs, it was only possible to identify the subfamilies (Kv1, Kv2, and Kv3). Thus, the presence and importance of Kv1 in HUASMCs make this subfamily of channels the main candidate for pharmacological studies in the fight against vascular diseases, mainly the hypertensive disorders of pregnancy. In this sense, the blocker 4-aminopyridine (4-AP) is the most used, but new activators of these channels have also been introduced to the market. Due to the importance of these channels, and the vast knowledge that exists about them, it is clear that modulation of Kv channels is a promising alternative for the treatment of vascular diseases, but it also plays a crucial role in the clinical practice of obstetrics and gynecology as a new pharmacological approach.

### 3.2. Calcium-Activated K^+^ (K_ca_) Channels

The K_Ca_ channels are important effectors in the control of vascular tone and blood pressure [33]. They mediate membrane hyperpolarization in response to elevations of intracellular Ca^2+^ and thus counteract SM contractility [34]. To date, three broad categories of K_Ca_ channels have been identified and classified according to their conductance [35]: large-conductance K_Ca_ channels, 100–300 pS (BK_Ca_) [36], intermediate-conductance K_Ca_ channels, 25–100 pS (IK_Ca_) [34,37], and small-conductance K_Ca_ channels, 2–25 pS (SK_Ca_) [34,38,39]. Each of them has important physiological and pathophysiological functions important to the CV system. BK_Ca_ channels are predominantly expressed in vascular SMCs and the inner mitochondrial membrane of cardiomyocytes. The activation of these channels leads to vasodilation and cardioprotection in situations such as cardiac ischemia. Concerning SK_Ca_, its channels can be largely found in the nervous and CV systems. Their activation is essential for the hyperpolarization of the cell membrane. The IK_Ca_ channels are expressed in SMCs and cardiac fibroblasts but are predominantly expressed in ECs and proliferating vascular SMCs. Their functions include proliferation, migration, vasodilation of SMCs, and cardiac fibrosis [35]. 

In the vascular SMCs, the dominant K_Ca_ channels are the BK_Ca_ channels (also designated BK, Slo, or MaxiK channels due to their primordial contribution to vascular tone control [40,41]. Its activation begins with the depolarization of the membrane and intracellular binding of Ca^2+^ and Mg^2+^ ions. On the other hand, these channels can also contribute to MP modulation in small myogenic vessels [5,16,20,42,43]. After the activation of BK_Ca_ channels, the cell membrane hyperpolarizes and triggers VGCC closure—a role that is opposite to that of these Ca^2+^-channels in vasoconstriction [41,44]. Some investigations have shown that the BK_Ca_ channels are activated by highly localized Ca^2+^ sparks produced by transient Ca^2+^-release events from the sarcoplasmic reticulum and/or Ca^2+^-influx through VGCCs. The resulting spontaneous transient outward currents (STOCs) produce hyperpolarization/repolarization and thereby counteract depolarization-activated VGCC activity. As a consequence of the VGCC inactivation, there is a decrease in [Ca^2+^]_i_, with consequence inactivation of myosin light-chain kinase, thus leading to relaxation [44,45,46,47,48]. 

Therefore, the BK_Ca_ channels have the function of regulating membrane voltage and [Ca^2+^]_i_, a function that is regulated by a diverse set of vasodilators and vasoconstrictors. In partnership with other ion channels, kinases, phosphatases, and regulatory proteins, the BK_Ca_ channels appear to be organized into macromolecular complexes that facilitate rapid and specific activation through different signal transduction pathways [41]. Some SM vasodilators, such as β_2_-adrenergic agonists, NO, prostaglandin I_2_, arachidonic acid, calcitonin gene-related peptide, and carbon monoxide, can activate these channels [5,48,49]. On the other hand, potent vasoconstrictors, such as angiotensin II, 5-HT, phenylephrine, and thromboxane A_2_, can inhibit them [48,49,50,51]. Overall, most agonist actions involve intracellular signaling pathways that include (1) Ca^2+^ sparks, which appear to be involved in vasodilator action through a negative feedback mechanism, (2) kinases that phosphorylate the channel protein, and (3) G-proteins that regulate channel activity independently of downstream kinases [48,49]. It has also been shown that BK_Ca_ channels can play a contributory role in altering the vascular tone in pathophysiological states (e.g., hypertension, stroke, atherosclerosis, diabetes, and CV surgery complications), and that is why they are also possible therapeutic targets in the treatment of these CVDs [35,41,52,53].

At the structural level, the examination of BK_Ca_ channel protein has been carried out by both X-ray crystallography and cryoelectronic microscopy [54,55,56]. These channels have a pore-forming α-subunit and a regulatory β-subunit [20,39,57]. The α-subunits contain six membrane-spanning domains (S1–S6), including voltage sensing (S1–S4; designated as VSD) and pore-gate (S5–S6, designated as PGD) domains [58]. However, the α-subunits, which are produced from a single gene (slo) by alternative splicing [20,59,60], contain an additional seventh transmembrane region (S0) that confers the N-terminus to the extracellular side and a large cytoplasmic domain containing ~800 amino acids [48,58]. Moreover, there are four β-subunit isoforms (β1–4), each with two transmembrane domains that may be associated with the α-subunits in a 1:1 ratio [43,61,62]. Among the four β isoforms, the β1 subunit is predominant in vascular SM [20,43,63,64]. The major function of the β-subunits is to enhance the Ca^2+^ sensitivity of the channel, and thus increase STOC activity and repolarizations and thereby facilitate relaxations [35,44]. The properties of the channels can be further modified by association with γ-subunits [52,65]. Concerning SK_Ca_ and IK_Ca,_ the main difference with the BK_Ca_ channel is that the Ca^2+^ sensor of SK_Ca and_ IK_Ca_ channels is calmodulin (CaM), which is constitutively bound to the calmodulin-binding domain (CaM-BD) in the C terminus and thus functions as a β-subunit that endows these channels with Ca^2+^ sensitivity [34].

At the pharmacological level, BK_Ca_ channels depend on their association with auxiliary subunits. The BK_Ca_ blockers are TEA (<1 mM), charybdotoxin, iberiotoxin, penitrem A, and paxillin, but only the last three compounds are selective for the BK_Ca_ channels [1,39]. On the other hand, these channels can be activated by agents that are called BK_Ca_ openers or BK_Ca_ activators. The BK_Ca_ openers comprise a large series of synthetic benzimidalone derivatives (e.g., NS1619 and NS004), biaryl amines (e.g., mefenamic and flufenamic acids), biarylureas (e.g., NS1609), pyridyl amines, natural modulators (e.g., resveratrol and dedydrosoyasaponin-1 (DHS-1)), and flavonoids [39,52]. Concerning SK_Ca_ and IK_Ca_, the neurotoxin apamin, a fully selective inhibitor of SK_Ca_ and IK_Ca_, can be blocked by charybdotoxin and the small molecule TRAM-34 [52]. 

Regarding the presence of K_Ca_ in HUA, Radenkovic et al. (2007) demonstrated that the SK_Ca_ channels appear to be present and functional at HUA [66]. However, Martin et al. (2014) suggested that SK_Ca_ appears to be in endothelial cells (ECs) and not SMCs as they did not find any small or intermediate conductance activated by an increase in (Ca^2+^)_i_ [15]. However, if SK_Ca_ channels are present in HUASMCs, their role does not seem to be very relevant since NO-induced relaxation did not activate these channels [31], and they also did not contribute to I_K_ in HUASMCs [18,23]. Regarding the role of IK_Ca_ channels in HUA, their presence has not been demonstrated. On the other hand, BK_Ca_ are fundamental in regulating the contractile state of HUA. Milesi et al. (2003) were among the first authors to demonstrate the presence of these channels in HUA. The authors demonstrated that TEA (5 mmol/L) induced contraction of HUA, and that chloretin (50µmol/L), an activator of BK_Ca_ channels, induced relaxation of them [18]. According to this investigation, other authors have also suggested the involvement of BK_Ca_ in the relaxation of HUA induced by NO [31], testosterone [21,23,67], levosimendan [32], and (-)-carveol [68] since this relaxation was inhibited by TEA. Additionally, Lovren et al. (2000) [31] demonstrated that BK_Ca_ channels are activated by NO at the single-channel level through a mechanism that probably involves PKG activation [69]. This hypothesis was confirmed years later by Cairrao et al. (2010), who demonstrated that the relaxation induced by testosterone in HUA is due to the opening of the BK_Ca_ channels through PKG-dependent action that is activated by increasing the intracellular levels of cGMP [23]. The electrophysiological experiments performed by the authors demonstrated reductions in I_K_ in the presence of TEA 100 µmol/L (35.9%) and iberiotoxin 0.1 µmol/L (36.4%) [23]. These findings are also supported by electrophysiological studies in fresh HUASMCs, in which reductions in I_K_ in the presence of 1 mmol/L TEA (±71%), 200 nmol/L iberiotoxin (±65%) [18], and paxilin 500 nmol/L [70] (±85%) were observed. The characterization of BK_Ca_ properties at the single-channel level was also in line with the results of previous investigations [18,70]. Thus, these results show that I_K_ in HUASMCs are mainly constituted by K^+^ exit through BK_Ca_ channels, together with Kv channels [15,23] (Figure 2). Overall, the authors found that the BK_Ca_ channels have all the biophysical properties typical of these channels in other tissues (for example, high selectivity to K^+^ or an increase in the probability of opening due to membrane depolarization and increased (Ca^2+^)_i_ [15].

In summary, there are some differences between the presence of the K_Ca_ channels in SMCs and HUASMCs. Concerning the SK_Ca_ channels, they are present in SMCs [35] and HUA. However, it is not yet known whether the SK_Ca_ channels are in ECs or SMCs of the artery, but the evidence seems to point to a location at the endothelial level. Regarding IK_Ca_ channels, the investigations demonstrate the presence of these channels in proliferative SMCs [35], but there is no scientific evidence in the literature to report their presence in HUASMCs. In contrast, concerning BK_Ca_ channels, the studies clearly demonstrate the presence of these channels in SMCs and HUASMCs, and their role in regulating vascular tone is well established. While the mechanisms by which BK_Ca_ channels contribute to vascular dysfunction are still unclear, on the other hand, their role in clinical practice is well known. In this sense, the BK_Ca_ channels are the more promising type of K_Ca_ channels that can be used as pharmacological targets for the prevention or reversal of depolarization, contraction, and maintenance of resting tone. Like Kv channels, there are several blockers identified and the modulation of these channels can be a promising alternative for the treatment of vascular diseases. At HUA, there is no certainty about the presence of SK_Ca_ and IK_Ca_. However, if the presence of these channels is proven in HUA, it is possible that their physiological relevance will not be significant and, therefore, their pharmacological importance will be small. Thus, in the development of a new pharmacological approach, BK_Ca_ channels should be the main therapeutic target to be used, for example, for the treatment of hypertensive diseases of pregnancy.

### 3.3. Inward Rectifier K^+^ (Kir) Channels 

The inward rectifier K^+^ (Kir) channels are a “special” class of K^+^ channels that can regulate an immensity of physiological processes since they control MP of excitable and nonexcitable cells [71]. At the functional level, these channels act as biological diodes as they are the only ones that can mediate the inward K^+^ flow by hyperpolarizing membrane voltages more quickly than the outward K^+^ flow at depolarizing voltages [72]. For a driving force of a given magnitude, the inward flow of K^+^ ions is greater than the outward flow. Hence, in these channels, the K^+^ flow can enter the cell (K^+^ circulates in the opposite direction). In this sense, the functional role of the Kir channels is critically dependent on their degree of rectification [7,73,74]. However, other K^+^ channels can also carry inward currents when E_m_ < E_K_, just like Kir channels. These channels are not activated by MP and are not controlled by voltage-gated channels. Instead, its activation is dependent on the difference between MP and E_K_ [7,75]. Some studies have strongly pointed out that the physiological role of these channels in vascular SM is related to MP maintenance and resting tone in small diameter coronary and cerebral arteries [76]. Kir channels were first described about 30 years ago in vascular SMCs as being present in only certain small-diameter cerebral and submucosal arterial SM and in coronary arterial SM [76]. 

Structurally, Kir channels have four subunits forming two transmembrane (TM) spanning helices, called TM1 and TM2, that are connected to the pore region containing the selectivity filter for K^+^ ions, with intracellular C- and N-terminal domains. [77,78]. TM1 (the outer helix) makes contact with TM2 (the inner helix), which lines the pore of the channel and forms an “inverted tepee” in a closed channel conformation [1,75,79]. These channels are encoded by members of the Kir gene family. The most relevant Kir expressed in the vasculature belong to the Kir2.x (strong inward rectifier), and K6.x (weakly inward rectifier) subfamilies [1]. Four isoforms of Kir2.x family have been identified, Kir2.1 to Kir2.4, however in vascular SMCs, only Kir2.1, 2.2, and 2.4 have been identified [20]. Kir2.2 has only been detected in cerebral arteries [80], and Kir2.4 expression has only been detected in SMCs of the human pulmonary artery [81]. In vascular SMCs, Kir2.1 is the dominant isoform expressed, particularly in mice cerebral, coronary, and mesenteric arteries [1,8,82]. Additional studies are required to characterize the expression and function of each Kir subtype.

At the pharmacological level, Kir channels can be blocked by extracellular Ba^2+^ and cesium (Cs^+^) ions, in addition to small molecule inhibitors being developed for specific channels. Modulation by other factors, including magnesium, extracellular K^+^ concentration, intracellular H^+^, ATP, phosphorylation by protein kinases A and C (PKA and PKC), and protein–protein interactions, is distinct to each Kir subfamily [1,8,78,83].

Regarding the Kir in HUA, V. Milesi has unpublished results that show that the blocker of Kir channels, Ba_2+_ (500 µmol/L), induces contraction of unstimulated HUA rings, which suggests that Kir channels may be involved in the maintenance of the basal contractile state of HUA. However, another study by Radenkovic et al. (2007) showed that the same blocker, Ba^2+^ (3 µmol/L), did not affect the top of bradykinin contractions of HUA rings coming from normal pregnancies, suggesting, on the contrary, that Kir channels are not involved in this mechanism [66]. However, the same authors found that this agent increased the bradykinin-induced strength in those HUA rings from pregnant women with gestational diabetes mellitus, which emphasized the clinical importance of the different K^+^ channels in pathological situations in pregnancy. More recently, Zhu et al. (2016) observed in SMCs from rat umbilical vein by patch-clamp that barium chloride did not affect I_K_ [84].

In summary, Kir channels are important in MP control, and their presence has been demonstrated in SMCs, with the Kir2.1 isoform being the most dominantly expressed. However, concerning the Kir channels in HUA, the studies seem to suggest their presence, but the studies carried out on HUA are very few, and, therefore, there is insufficient scientific evidence to classify these channels as potential pharmacological targets. Thus, further studies are needed to clarify its role in clinical practice. Since the initial approaches involved the use of the Ba^2+^ ion, it is suggested that other methodologies, such as protein kinase modulation, for example, be approached in future studies. 

#### ATP-Sensitive K^+^ (K_ATP_) Channels

The ATP-sensitive K^+^ (K_ATP_) channels were first identified in the cardiac muscle in 1983 [85,86]. After that time, the presence of these channels was also identified in other cells, including vascular SMCs [1,5,7,20,87,88,89], and its role has been studied at different levels, including CVDs [90]. The K_ATP_ channels belong to the Kir superfamily and conduct weak inward rectifier I_K_ [90]. Overall, K_ATP_ channels act as metabolic sensors and maintain homeostasis in acute metabolic changes, including hyperglycemia, hypoglycemia, ischemia, and hypoxia [91]. Therefore, the K_ATP_ channels are designated as having an ability to decrease its activity when the intracellular concentration of ATP increases. This activity, interestingly, is modulated by several signaling pathways that are independent of ATP sensitivity [7]. 

At the structural level, the K_ATP_ channels are a macromolecular complex (~900 kDa) consisting of 4 pore-forming subunits (Kir6.1 encoded by KCNJ8 or Kir6.2 encoded by KCNJ11) and 4 regulatory sulfonylurea receptor (SUR) ATP-binding cassette subunits (subfamily C: SUR1, SUR2A, or SUR2B). Different combinations of Kir6.1 or Kir6.2 and SUR1 or SUR2 variants (SUR2A or SUR2B) in cell lines constitute K_ATP_ channels with distinct electrophysiological and pharmacological properties that correspond to the various K_ATP_ channels in native tissues [91]. In vascular SM, the combination Kir6.2/SUR2B is likely the most widespread, although Kir6.1/SUR2B may also be present in this tissue [92,93]. K_ATP_ channels are inhibited by ATP and ADP (channels close) and activated by Mg-ADP and Mg-ATP (channels increase activity), allowing the cell to engage the cellular metabolic state (ATP/ADP ratio) to electrical activity of the cell membrane [90,94].

At the pharmacological level, K_ATP_ channels are blocked by antidiabetic sulfonylurea drugs such as tolbutamide, glibenclamide, and glimepiride [95]. Glibenclamide is the most frequently used inhibitor in studies performed in arterial SMCs and tissues. Some artery contractility studies have elucidated that the half-inhibitory concentration of glibenclamide oscillates between 20 and 200 nM [5,20], and, for tolbutamide, the half-inhibitory concentration is 350 µmol/L [20]. External Ba^2+^ can also block the K_ATP_ channels, with a half-inhibitory concentration of 100 µmol/L at –80 mV [5,20]. Furthermore, 4-AP also induced a small inhibition (10%) at 1 mmol/L [1]. Several antihypertensive drugs seem to act through K^+^-channel activation and have been designated as K^+^-channel openers. These openers exhibit extreme chemical diversity and comprise a few different structural classes. The most used are nicorandil, cromakalim, pinacidil, minoxidil sulfate, aprikalim, diazoxide, and lemakalim [95].

Regarding the K_ATP_ in HUA, currently, there is still no consensus of their participation in the contraction/relaxation of intact HUA rings [15,21,66]. Several studies have shown that the K_ATP_ blocker glibenclamide (1 and 10 µmol/L) did not affect HUA rings when applied on top of bradykinin [66], histamine, KCl, or serotonin-induced contractions [21]. Moreover, glibenclamide (10 µmol/L) did not modify NO- or levosimendan-induced relaxations, ruling out the participation of K_ATP_ channels in such contractile and relaxing mechanisms [31,32]. In contrast, glibenclamide partially impaired relaxation in the human umbilical vein (HUV) rings caused by sodium sulfide (Na_2_S), suggesting that part of the relaxation is due to the increased open time of the K_ATP_ channels [96]. Concerning I_K_ in HUASMCs, it has been reported by whole-cell patch-clamp techniques that K_ATP_ channels do not contribute to the total current because glibenclamide does not affect I_K_ [23]. Furthermore, Zhu et al. (2016) also observed on SMCs from rat umbilical vein by patch-clamp that glibenclamide did not affect I_K_ [84]. In contrast, Bai et al. (2013) noted that when using a high K^+^ concentration (140 mmol/L) and perfusing a K^+^-channel-specific opener, pinacidil, the I_K_ increased [97]. More recently, Li et al. (2018), using the whole-cell patch-clamp technique, also demonstrated in HUASMCs that the application of pinacidil (10 µmol/L) increased I_K_, but glibenclamide (10 µmol/L) completely inhibited the K^+^ resulting from the application of pinacidil [88]. The authors also identified mRNA expression levels of Kir6.1, Kir6.2, and SUR2B subunits in these cells [88]. 

In summary, K_ATP_ channels play an important role in regulating vascular tone, and their presence has been demonstrated in SMCs and HUASMCs. Moreover, differences in subunits were not found since, in both cell types, levels of mRNA expression of Kir6.1, Kir6.2, and SUR2B subunits were found. At the pharmacological level, the blocker glibenclamide is the most used, including in studies on HUA. However, the role of K_ATP_ channels in the physiological regulation of HUA is not clear, and, therefore, the role of these channels in clinical practice is still unknown. More pharmacological studies are needed, but it is assumed that due to little physiological relevance, the role of K_ATP_ at the pharmacological level will not be relevant.

### 3.4. The 2-Pore Domain K^+^ (K_2P_) Channels 

The 2-pore domain K^+^ (K_2P_) channels were first identified in the late 1990s [98,99]. This channel family produces leak-type K^+^ currents [100] and are involved in controlling and stabilizing MP and the levels of cellular excitability [1,100,101].

Structurally, K_2P_ channels are characterized by having two pore-forming regions (P1–P2) in each channel subunit, surrounded by four transmembrane-spanning (4TMS) helices and a large “cap” domain. Moreover, these channels are different from the remaining K^+^ channels because they are the smallest, and they do not form tetrameters but functional dimers [1,100,102]. The K_2P_ channels elicit spontaneously active and outwardly rectifying “background leak”-type K^+^ conductance. However, these channels have no classical time-dependent or voltage-dependent activity [103,104,105,106,107,108]. 

Currently, in mammals, 15 different K_2P_ channels have been described/identified, each with important physiological roles at different levels. These 15 channels can be divided into six families, according to their biophysical and pharmacological properties [103,104,106,107,109]. The TWIK2 (tandem of P-domains in a weakly inward rectifying K^+^ channel, K_2P_6.1), TASK-1 (TWIK-related acid-sensitive K^+^ channel, K_2P_3.1), and TREK-1 (TWIK-related K^+^ channel) families are the ones that presented the highest levels of expression in SMCs [110,111,112]. Unlike most K_2P_, TWIK2 are highly expressed in all blood vessels studied so far (including resistance-sized vessels), which makes K_2P_6.1 the main candidate for physiological regulation in the vascular system [111,113]. On the other hand, the contribution of TASK-1 to the regulation of vascular tone is less understood, and it, therefore, needs further investigation [110,114,115].

At the pharmacological level, the K_2P_ channels are very weakly sensitive or insensitive to classical K^+^ channel blockers (e.g., TEA, Ba^2+^, Cs^+^, and 4-AP). Instead, two external blockers of these K^+^ channels in arterial SM, anandamide 10 µmol/L [114] and zinc ion (Zn^+2^) 100 µmol/L [114,116], and two openers, halothane [114] and ONO-RS-082 [117], have been reported. Moreover, these channels are sensitive to important biophysical stimuli (e.g., pH, temperature, and mechanical pressure), and some of them are inhibited by the acidification of extracellular fluid [1,100,116,118]. The K_2P_ channels are also targets for drugs (including inhalational and local anesthetics, antipsychotics, antidepressants, and neuroprotective agents), and modulated by G protein signaling pathways and second messengers [103,104,106,107,119,120]. Alternate splicing [121,122,123,124], alternative translation initiation [125], and heterodimerization of K_2P_ channel subunits [126,127,128] make studying these channels even more complex.

To our knowledge, there is no definitive evidence concerning the presence of K_2P_ channels in HUA, although their presence has already been demonstrated in other vessels. However, the fact that there are no specific blockers for these channels makes it difficult to study them in more detail [15]. Martin et al. (2014) reported detecting mRNA for TRAAK and TREK1 in HUASMCs but stressed the need for functional and more specific characterization to confirm its presence. In the same sense, it has been hypothesized that K_2P_ channels are also present in placental vessels, but the evidence is still preliminary [15]. Wareing et al. (2006) detected TASK1 mRNA in the arteries of chorionic plaque and a possible blockade of the channels with anandamide, but these authors also concluded the need for further investigations to be developed, mainly due to the nonspecificity of the compound [129]. 

In summary, the K_2P_ channels are the most recently identified channels in SMCs. Its physiological role in SMCs is to control and stabilize MP and levels of cellular excitability. Members of the subfamilies of weak inward rectifiers (TWIK-2), acid-sensitive rectifiers (TASK-1), and lipid sensitive mechano-gated channels (TREK-1) have been identified in SMCs. However, in HUASMCs, only the existence of TREK-1 and TRAAK, both belonging to the subfamily of lipid sensitive mechano-gated channels, has been suggested. The fact that there are no specific blockers for these channels makes it difficult to study them in more detail and prove their existence in HUA. In this sense, the next step will be the development of new studies to find specific blockers/activators for these channels. A focus on TREK-1 channels is suggested to find out if these channels can be a pharmacological target in the treatment of vascular diseases. 

## 4. Clinical Importance and Medical Uses of K^+^-Channels

Gestational hypertension and preeclampsia are hypertensive disorders of pregnancy [130] that affect 10% of all pregnancies in the world [131,132]. Hypertension is the most common medical problem encountered during pregnancy (6–20% of all pregnancies), and it is highly responsible for maternal and perinatal mortality and morbidity. In addition, it is associated with an increased risk of developing CVDs in pregnant women [13]. On the other hand, pre-eclampsia comprises 3–5% of hypertensive disorders in pregnancy. According to Fox et al. (2019), pre-eclampsia (PE) is defined as “new-onset hypertension after 20 weeks’ gestation, with evidence of maternal organ or uteroplacental dysfunction or proteinuria”. This disorder is a major cause of maternal morbidity and has many adverse effects associated with fetal health. There is evidence that the effects of PE go beyond fetal health and may be reflected in the long term by sequelae in the offspring’s CV system (such as hypertension and altered vascular function) [132,133]. 

As described above, K^+^-channels are extremely important for the control of HUA vascular tonus, regulating SMC membrane potential [1]. Due to its importance, it is not surprising that its molecular composition, activity, and expression are crucial parameters to know. Particularly, this issue assumes greater relevance when it comes to vascular pathologies or being associated with pregnancy, in which changes in the expression and function of these channels in SMCs have already been reported.

Starting with K_v_ channels, changes in the properties and functions of these channels in hypertensive vascular SMCs were reported [134,135]. These changes can lead to vasoconstriction that alters cellular mechanisms and culminates in hypertension [5]. Moreover, studies in mesenteric arteries of several hypertensive animals have shown that the expression of the α-subunit gene and the protein in these channels were elevated [134]. Additionally, studies performed in hypertensive vascular SMCs have shown that the K_v_ currents were lower than those in BK_Ca_ [136,137] and that this decrease had intracellular Ca^2+^ as the main player [134]. The same authors also demonstrated, by electrophysiological studies, that Ca^2+^ can be regulated by K_v_ channels during hypertension since an increase in protein expression seemed to be related to a decrease in functionality [136,137]. This decrease may underlie the subsequent depolarization of the membrane and, therefore, a consequent increase in vascular tonus observed in this pathology [16]. In the last year, Djokic et al. (2019) also demonstrated that during gestational hypertension, the function of the Kv channels was compromised as they observed an absence of inhibition of the functional response to pinacidil (K_ATP_ channel opener) [138]. The authors also demonstrated that 4-AP significantly decreases vasorelaxation induced by pinacidil in vascular SMCs of HUV from normotensive pregnant subjects, but not in hypertensive pregnant subjects [138]. Furthermore, the same authors reported in this year (2020) that in hypertension situations, there is a decrease in Kv1.3 channel expression and function in SM of HUV [139]. Concerning PE, studies in pregnancies complicated by uterine growth restriction have also shown that venous contractility, but not the arterial contraction of chorionic plaque arteries (CPA), was increased. The nonspecific Kv1-4 channel inhibitor (4-AP) induced contraction less effectively compared to normal pregnancies, which suggests that the activity of Kv channels is decreased in PE situations [140,141]. Indeed, studies have shown that Kv7 channels are involved in the regulation of vascular tone in CPA during normal pregnancy [142,143]. However, some authors have reported that the protein of the Kv channels (KCNQ3 and KCNE5) is significantly increased in the placenta of women with PE, while KCNQ4 and KCNQ5 appeared to be downregulated [142,144]. On the other hand, the loss of function of the Kv channels is linked to a series of lethal or debilitating channelopathies. New pharmacological approaches have been developed to selectively activate specific Kv channels [145]. In HUA, only the Kv1, 2, and 3 channels exist. However, currently, the only approved drugs are retigabine, an antiepileptic and anticonvulsant, which activates Kv7 channels in the brain, but which was discontinued in 2017 due to serious side-effects, including retention urinary, bluish skin, and retinal discoloration, and flupirtine, which also shows antiepileptic activity and acts by activating the selected Kv7 channels [146]. However, several other Kv7-specific activators are now available (see review in [146]). Regarding Kv1 channel openers, a selective activator is currently missing. However, acetazolamide, a diuretic inhibitor of carbonic anhydrase, and flupirtine, an analgesic and muscle relaxant, appear to induce the activation of Kv1 [1,147]. The Kv1 channel closers, like for the BK channels, are not specific, and the concentration determines their selectivity. The main blockers used are 4-AP (1 mM), charybdotoxin (0.1 µM), and TEA (5–10 mM) [1]. On the other hand, the inhibitor margatoxin was initially considered a selective inhibitor of the Kv1.3 channel, but recent data also show that it can inhibit Kv1.1, -1.2, and -1.7 [148]. Furthermore, 4-AP (also designated as dalfampridine) is an approved drug for improving motor function in patients with multiple sclerosis [149], and 3,4-diaminopyridine (also designated as amifampridine) is recommended as a first-line symptomatic treatment for Lambert–Eaton myasthenic syndrome [150]. However, Kv channel blockers are also approved as antiarrhythmic agents used to maintain normal sinus rhythm and cardioversion in cases of atrial fibrillation and atrial flutter (dofetilide, a Kv10 and Kv11 inhibitor) [151] and are used for the treatment of recurrent hemodynamically unstable ventricular tachycardia and recurrent ventricular fibrillation (amiodarone, a Kv1 inhibitor) [152]. In summary, due to the importance of Kv channels, namely, Kv1, and the vast knowledge that exists about them, it is clear that modulation of these channels is a promising alternative for the treatment of vascular diseases. As demonstrated, the impaired functional activity of the Kv channels contributes to hypertension. Thus, more detailed insight into the expression and function of the different Kv channels present in HUASMCs (Kv1, -2, and -3) can be an asset in understanding hypertensive diseases of pregnancy and help in the development of new strategies to reduce the outcomes in this stage of development.

Regarding the BK_Ca_ channels, studies have shown that during a stage of hypertension, there is an increase in the expression of these channels [153,154,155]. Studies performed on cerebral arteries of hypertensive rats have a decrease in BK_Ca_ activity when the expression of BK_Ca_ β1-subunit is decreased. However, changes in the BK_Ca_ α-subunit have not been found [156,157]. Thus, when the BK_Ca_ β1-subunit is deleted, there is a decrease in Ca^2+^ sensitization, and the channel stops responding to Ca^2+^ sparks. In this way, membrane depolarization begins, and intracellular Ca^2+^ increases, promoting vasoconstriction and increased blood pressure, which culminates in hypertension [57]. Further studies by Ledoux et al. (2006) demonstrated that changes in the channel conformation or sensitization to Ca^2+^ can lead them to act as pure K_V_ [57]. Like K_v_, the mechanism responsible for the currents of BK_Ca_ channels during hypertension seems to occur through negative feedback: the protein expression of the channels increases in response to an increase in vascular tonus [16,137]. Radenkovic et al. (2007) demonstrated that the role of BK_Ca_ channels is different in the force exerted by bradykinin between HUA of normotensive and hypertensive pregnancies, as demonstrated by an increase in bradykinin contractions induced by TEA in this latter case [66]. In agreement with these results, Eichhorn et al. (2007) observed an increase in Ca^2+^ flux and BK_Ca_ activity in SMCs of different hypertensive animals. However, these authors, contrary to others, observed an increase in the expression of both subunits (α and β) of BK_Ca_ [41]. In the same study, it was possible to observe that the inhibition of BK_Ca_ channels by specific blockers depolarizes the membrane and gives rise to vasoconstriction [41]. Recently, the regulated trafficking of BK channel subunits (including α, β1, and γ subunits) has been accepted as a functional mechanism to modulate arterial contractility. When β1 trafficking is reduced, there is a decrease in BK channel currents and vasoconstriction observed in hypertension [158,159]. In some studies on animal models of hypertension, where there was a treatment with BK_Ca_ channel blockers, exaggerated vasoconstrictor responses were observed, supporting the hypothesis that there is an increased activity of these channels in the presence of the pathology [42,48]. Joseph et al. (2013) also reported that iberiotoxin-sensitive I_K_ (a selective blocker of BK_Ca_ channels) were elevated in hypertensive animals compared to control animals [160]. Accordingly, Djokic et al. (2019) also suggested that hypertension leads to positive regulation of BK_Ca_ as a compensatory mechanism for blood vessel hyperactivity. However, as there were no changes in the MaxiK-α subunit, this regulation does not appear to be the result of an increase in the number of channels. This regulation is likely to be the result of post-transcriptional modifications of the MaxiK-α subunits and/or changes in interaction with auxiliary subunits [138]. Concerning PE, He et al. (2017) reported that BK_Ca_ channel impairment in human CPA is potentially relevant to the development of the pathology. Specifically, the authors found that decreased expression or activation of these channels induces a pathological CPA remodeling. This process weakens vasodilation and decreases the sensitivity of the artery to vasoactive substances; consequently, there is a decrease in fetoplacental blood flow that can induce PE [161]. Also, in pre-eclampsia, NO and IK_Ca_ and SK_Ca_ channels play an important role in the vasodilation of CPA [162]. Li et al. (2017) demonstrated that these channels are in the endothelium and SM of CPA and that women with PE presented unregulated levels of expression compared to normotensive women. The expressions of endothelial (eNOS) and inducible (iNOS) oxide nitric synthase were also subregulated, which was reflected in a decrease in eNOS activity. Thus, the authors suggested that during PE, IKCa- and SKCa-mediated vasodilation was impaired and that these channels are responsible for NO-mediated vasodilation and modulation of NO synthase activity. The authors concluded that the deregulation of these channels may be involved in the pathogenesis of PE, directly promoting vascular constriction of CPAs and affecting NO functions and NOS activities [162]. As demonstrated in Section 3.2., the presence of SK_Ca_ and IK_Ca_ channels in HUA is uncertain. Thus, in the development of a new pharmacological approach, BK_Ca_ channels should be the main therapeutic target used. At the pharmacological level, BK_Ca_ channel openers can be classified based on their origin and structure in (A) endogenous BK_Ca_ channel modulators and structural analogs, (B) naturally occurring BK_Ca_ channel openers and structural analogs, and (C) synthetic BK_Ca_ channel openers. Endogenous chemicals such as arachidonic acid and the metabolites of cytochrome P450, epoxygenase, and lipoxygenase have been found to increase BK_Ca_ channel activity and be important regulators of vascular tone. Natural occurring BK_Ca_ channel activators, such as terpene derivatives (e.g., pimaric acid and maxikdiol), flavonoids (e.g., apigenine, naringenin), and phenolic derivatives (e.g., magnolol), may be found in herbs, roots, fungi, and leaves, and have been used in folk medicine for the treatment of asthma and smooth muscle disorders. Synthetic BK_Ca_ channel activators capable of increasing channel-open probabilities have been synthesized by several companies, such as benzoimidazolone derivatives (e.g., NS1619, NS 1608, and NS004), carboxylate compound CGS7181 and its analogues (e.g., CGS7184), 1,4-benzothiazine derivatives, and NS11021 [163]. Of these, NS1619 showed that it may prevent ischemia-reperfusion-induced inflammation [164], pressure overload-induced heart failure, and decreased coronary vasodilatory capacity in heart failure with preserved ejection fraction patients [165]. The BK_Ca_ channel closers are the scorpion toxins (charibdotoxin and iberiotoxin) and tetraethylammonium (TEA); however, these BK_Ca_ inhibitors are not specific to these channels, the concentration being extremely important to make them quite specific (TEA, K_Ca_ inhibitor, 5 mM; charybdotoxin (ChTx), BK_Ca_ inhibitor, 10 nM; iberiotoxin (IbTx), BK_Ca_ inhibitor, 0.1–100 nM) [1]. Thus, in the development of new pharmacological studies in HUA, the BK_Ca_ channels are, with Kv channels, the main therapeutic targets to be used in the treatment of hypertensive diseases of pregnancy.

Concerning the Kir channels, the presence of these channels in HUA is still uncertain. However, at the pharmacological level, the targeting of Kir channels could be a great strategy to induce hyperpolarization/vasodilation and to reduce the peripheral resistance and high blood pressure observed in hypertension. Watanapa et al. (2012) observed inhibition of the Kir expression by PE plasma in HUVEC cultures. The same authors also observed the expression of K_Ca_ that seems to be a compensatory mechanism for the Kir reduction. In hypertension, it has also been observed that K_IR_ channels have their expression or function diminished [42]. Tajada et al. (2012) also demonstrated a decrease in Kir2.1, Kir4.1, Kir6.x expression, and a functional decrease in these channels [166]. These findings point out the Kir channels play an important role in the hypertensive disorders and act with a pharmacological target. As the Kir2.1 isoform is the most dominant one expressed in SMCs, pharmacological studies focusing on this subunit are of great interest. The Kir channel opener flecainide, an antiarrhythmic drug, increases Kir2.1 currents by binding to cysteine 311 and reducing polyamine-induced rectification [167]. To our knowledge, this drug is a unique drug that can activate Kir 2.1. channels [168]. Moreover, the ion Mg^2+^ was proposed as an activator of Kir channels in vascular SMCs [1]. In contrast, several drugs are proposed as blockers of Kir channels, namely, the antimalarial drug chloroquine [169,170], the antiarrhythmic drug quinidine [167], the diamine pentamidine analog 6 (PA-6) [171], and a more recent prototype of Kir blocker, ML133 [172]. Moreover, these channels are markedly sensitive to inorganic cations (e.g., Ba^2+^ and Cs^+^) but are insensitive to 4-AP [168]. Although the presence of Kir channels in HUA is uncertain, the next pharmacological studies focusing on the development of new targets for Kir2.1 channels can be the first promising step at the vascular level.

Concerning the K_ATP_ channels, studies have also reported their involvement in gestational pathological processes such as gestational hypertension [138]. Changes in the function and expression of these channels have already been reported in vascular SMCs of various vascular beds and different species in the presence of this pathology [7,138]. The reduction in the function of the K_ATP_ channels has been implicated in hypertension. Several studies have shown reduced relaxation of SMCs in various types of arteries when applying agonists [166,173,174,175,176,177] of the K_ATP_ channels or openers [178,179] of the same. In 2005, Gutterman et al. reported that the vascular oxidative state is regulated by physiological stimuli or pathophysiological stress and includes hypertension [180]. Indeed, the presence of chronic hypoxia or elevated oxidative stress has been demonstrated in situations of gestational hypertension and, thus, can compromise the health of the mother and fetus [181,182,183,184]. Many researchers have suggested that K_ATP_ channels are key agents in hypoxia-mediated vasodilation since hypoxia affects the ATP/ADP ratio but also because, when the activity of the channels is impaired, this process is attenuated [185,186]. Thus, it seems that the K_ATP_ channels can be excellent pharmacological targets in the treatment of pregnancy disorders such as gestational hypertension. In addition, it is described that the coexpression of both subunits of the K_ATP_ channels, in the ratio 4:4, is fundamental for their normal functional response [78]. On the other hand, the gain in Kir6.1 subunit function with the hyperactivity of these channels leads to hypotension, a phenomenon that has been confirmed in people with Cantu syndrome [187]. Djokic et al. (2019) observed in HUV that in situations of gestational hypertension, there was a decrease in the expression of the Kir6.1 pore-forming subunit, while the expression of SUR2B remained unchanged. Exposure of a too-short HUV to the hypertensive environment or the therapeutic application that maintains vascular tension during pregnancy may be possible explanations for the unchanged expression of SUR2B [138]; not least because a previous study carried out with SM of the aorta of spontaneously hypertensive rats demonstrated that as the exposure time increased, the decrease in expression observed for this subunit was increased [188]. However, Tajada et al. (2012) reported a reduced expression of SUR2B in their investigation, as well as a decrease in the function of K_ATP_ channels that impaired vascular tone [166]. In this sense, it has been suggested that hypertension can be reversed by restoring the K_ATP_ channels [178,189]. Du et al. (2019) also demonstrated that the Kir6.1 subunit was negatively regulated in the myometrium of parturient suffering from gestational hypertension. The authors suggest that since the K_ATP_ channels are responsible for uterine stillness in pregnancy, this regulation could explain the risk of premature labor associated with parturients with hypertension [190]. Thus, it seems plausible to say that in the presence of gestational hypertension, there is a decrease in the expression of K_ATP_ channels in SMCs [7,138]. However, despite the decreased expression of K_ATP_ channels observed in SMCs, Djokic et al. (2019) have found no changes in pinacidil-induced relaxation (K^+^ channel opener) in the presence of glibenclamide in situations of gestational hypertension in humans, and similar results have also been obtained in spontaneously hypertensive rats [191]. The fact that pinacidil completely relaxes HUV in the presence of glibenclamide supports the hypothesis that it acts partially through mechanisms independent of K_ATP_ channels, mainly in high doses, activating another type of K^+^ channel (such as BK_Ca_ and K_v_) [138,192,193,194,195]. On the contrary, some studies have reported that there were no changes in the functions of the K_ATP_ channels [191,196,197] or the functions being improved [198,199] in hypertensive models. Recently, Tykocki et al. (2017) pointed out in their review that hypertension may be the cause of the deregulation of these channels observed in hypertensive models [7]. However, due to the fact that the information available is not fully agreed upon, further studies are needed to understand the expression and function of the K_ATP_ channels in hypertension. Furthermore, since the K_ATP_ channels are present in HUA, the knowledge about the role of these channels is crucial to improving the treatment of hypertensive disorders of pregnancy. At the pharmacological level, K_ATP_ channel openers are chemically diverse and belong to several structural classes. These include benzopyrans (levcromakalim, bimakalim), benzothiadiazines (diazoxide), cyanoguanidines (pinacidil), cyclobutenediones (WAY-151616), nicotinamides (nicorandil), pyrimidines (minoxidil), tertiary carbonoles (ZD-6169), thioformamides (aprikalim), and dihydropyridine-like structures (ZM-244085) [200]. Some of these drugs, like diazoxide, pinacidil, minoxidil, nicorandil, and cromakalim, have been used to treat hypertension, myocardial ischemia, bronchial asthma, urinary incontinence, hyperinsulinism, angina pectoris, and some forms of skeletal muscle myopathies. However, some have been withdrawn due to their adverse effects: for example, the vasodilation caused by this mechanism resulted in reflex tachycardia and fluid retention, which oppose the antihypertensive effect. In contrast, K_ATP_ channel closers stimulate insulin secretion and, therefore, pharmacologic closers like glibenclamide are one of the only oral medicines available to treat diabetes. More recently, the development of K_ATP_ channel closers called PNU compounds (PNU-37883A, a morpholinoguanidine, and PNU-99963, a cyanoguanidine) have been shown to inhibit the vasodilation and hypotension caused by traditional K_ATP_ channel openers [201]. More studies are needed to improve the knowledge of K_ATP_ channels in HUA and unravel how these channels can be a good target in the treatment of hypertensive diseases of pregnancy.

Concerning the K_2P_ channels, the presence of these channels in HUA is uncertain, and only Martin et al. (2014) have reported the presence of mRNA from the TREK-1 and TRAAK channels. Increasing evidence shows a role for TREK channels in cardiac pathologies such as atrial fibrillation and heart failure [202,203,204]. TREK-1 channel openers include riluzole, chloroform, and diethyl ether. Moreover, these channels are opened by volatile general anesthetics such as halothane and isoflurane [103]. New synthetic compounds have been developed to modulate these channels namely, GI-530159, a new activator of the TREK1 (but not TRAAK) channels [205] and BL-1249 [206], which, in addition to directly activating TREK-1 channels, activates other channels such as TREK-2, TASK3, BKCa, KV7.2, KV7.3, and NaV1.7 channels [205]. Moreover, a dihydroacridine analog (ML67-33) has been described to selectively activate the TREK-1, TREK-2, and TRAAK channels, and has no effect on the TASK1, TASK2, and TASK3 or KCNQ2 channels [207]. Additionally, TREK-1 channels are blocked by quinidine and reversibly blocked by Gd^3+^ at micromolar concentrations. In contrast, these channels are resistant to TEA and 4-AP and slightly sensitive to high concentrations of Ba^2+^. In terms of physiological regulation, TREK-1 (but not TRAAK) channels are inhibited by activators of PKA and PKC [103]. Recently, Cunningham et al. (2018) have demonstrated that treprostinil, a stable prostacyclin analog, has a direct inhibitory effect on TREK-1 channels. These results are quite promising because treprostinil is commonly used in the therapy of pulmonary arterial hypertension to keep blood vessels open [208]. Indeed, there are relatively few selective antagonists for the TREK channels. Some examples of blockers include chlorpromazine, sipatrigine, fluoxetine, and norfluoxetine [209]. However, there is currently a highly specific and fast-acting inhibitor on TREK-1 channels, spadin. This drug does not affect the TREK-2 or TRAAK channels and acts by promoting a decrease in currents by internalizing the TREK-1 channel [210]. In clinical practice, the antiarrhythmic drug diltiazem inhibits TREK-1 (but not TRAAK) channels [211] and is used to treat hypertension and angina pectoris. Moreover, TREK channels are also modulated by other cardiac-related drugs, e.g., mibefradil [119] and quinidine [212]. Taken together, these results suggest that TREK-1 channels are promising therapeutic candidates for the development of new pharmacological studies in HUA to be used in the treatment of gestational hypertension. Concerning TRAAK channel openers, riluzole [213] and ML67-33 [207] activate these channels. On the other hand, these channels are reversibly blocked by micromolar concentrations of Gd^3+^, but resistant to TEA and 4-AP, and slightly sensitive to high concentrations of Ba^2+^ [103]. The drugs sipatrigine [214] and pimozide [215] inhibit TRAAK channels, but several other drugs tested (e.g., fluoxetine, paroxetine [216], lamotrigine [214], chlorpromazine [217], haloperidol, sulpiride [215], and diltiazem [211]) had no effect on them. Due to the fact that pharmacological knowledge of these channels is still very premature, more studies are needed to investigate the role of TRAAK channels as pharmacological targets in the treatment of hypertensive diseases of pregnancy.

In summary, the study of the physiological regulation of K^+^ channels present in HUA is crucial to detect potential targets for the treatment of pregnancy-related pathologies [9]. Gestational hypertension and pre-eclampsia are multifactorial disorders and are important risk factors in the development of CV complications that endanger not only the health of the mother but also the health of the fetus. As evidenced, studies on the UC vasculature and, in particular, HUA are practically scarce, even though morphological changes have been associated with these pathologies [13]. Thus, further studies are needed to understand what mechanisms underlie these pathologies, namely, with concern to the involvement of HUA K^+^ channels. As mentioned, there are several K^+^ channel activators and inhibitors that could be used in the treatment of hypertensive disorders, but some challenges remain to be overcome. One of these is the use of inhibitors that induce contraction, which is hard to reconcile with treating hypertension. However, there may exist an interaction between the various types of channels since the blocking of one type of K^+^ channel can activate another K^+^ channel, and vice versa; for example, it was observed with 4-AP that it can enhance Kv7.4 channel activity [149]. Moreover, a very important additional issue is the selectivity; most of the compounds are nonselective, and they act on a wide range of channels. Furthermore, since the K^+^ channels are present in many types of cells, the likelihood of adverse effects on other organs increases. Therefore, achieving specific targeting is an extremely challenging task.

## 5. Conclusions and Future Perspectives

HUA is an excellent sample for obtaining human vascular SMCs, allowing the study of different cellular mechanisms and their functions. The study of its physiological regulation is extremely important because as HUA does not have nerve endings, the regulation of its vascular tone depends completely on other factors, where the K^+^ ion is included. Moreover, K^+^-channels are one of the main mechanisms involved in the vasorelaxation of this artery. Indeed, the K^+^-channels are fundamental in determining and/or modulating the membrane potential, allowing them to regulate the vascular tone of the artery. The functional presence of the Kv and BK_Ca_ channels is clearly demonstrated in HUA, and its function of regulating the contractile state is also well established. Concerning the SK_Ca_, Kir, K_ATP_ and K_2P_ channels, their presence is also suggested, but there are still very few studies and, therefore, much of the information about their functions remain unknown. On the other hand, regarding the IK_Ca_ channels, we found no evidence in the literature to demonstrate its presence in the artery. In this sense, new studies are needed, not only focused on the expression but also the functionality of the different K+ channels of HUA. Several vasodilators and vasoconstrictors can act as modulators of these channels, and it is crucial to understand in detail the pathways by which the different K^+^-channels are regulated and the interaction that exists between them. These studies include determining the structure of the channel, the role of different subunits, and the molecular basis of regulating channel activity by different intracellular pathways. On the other hand, the localization of the different types of channels also plays an important role in the understanding of each one at different vascular levels. In this line of thought, genetic and molecular studies are suggested, complemented with electrophysiological studies. It is important to note that, as demonstrated, K^+^ channels are clinically relevant, particularly in hypertensive diseases of pregnancy (gestational hypertension and pre-eclampsia). Thus, the identification of changes in the expression and/or functionality of the different K^+^ channels present in these disorders can be an asset when it is intended for developing new drugs for their treatment. The study of HUA K^+^-channels and the signaling pathways involved in regulating artery contractility may contribute to the development of new specific drugs that inhibit or activate these channels, which can be used as therapeutic drugs or pharmacological tools. The complexity of the different combinations of K^+^ channels allows the vascular response to be directed to the most appropriate pharmacological treatment, depending on the pathology, which is why the study of K^+^ channels is so important and promising, as it represents an open window on the progression and enrichment of the knowledge of human vascular physiology.

## Abbreviations

(Ca^2+^)i	intracellular Ca^2+^ concentration
4-AP	4-aminopyridine
ANP	atrial natriuretic peptide
Ba^2+^	barium ion
BK_Ca_	large-conductance Ca^2+^-activated K^+^ channels
Ca^2+^	calcium
CaM	calmodulin
CaM-BD	calmodulin-binding domain
cGMP	cyclic guanosine monophosphate
Cl^-^	chloride
CPA	chorionic plaque arteries
Cs^+^	caesium ion
CV	cardiovascular
CVD	cardiovascular diseases
DHS-1	dedydrosoyasaponin-1
EC	endothelial cells
EK	K^+^ equilibrium potential
eNOS	endothelial oxide nitric synthase
HUA	human umbilical artery
HUASMC	human umbilical artery smooth muscle cells
HUV	human umbilical vein
I_K_	K^+^ currents
IK_Ca_	intermediate-conductance Ca^2+^-activated K^+^ channels
iNOS	inducible oxide nitric synthase
K^+^	Potassium
K_2P_	2-pore domains K^+^ channels
K_ATP_	ATP-sensitive K^+^ channels
K_Ca_	Ca^2+^-activated K^+^ channels
Kir	inward rectifier K+ channels
K_V_	voltage-dependent K+ channels
MP	resting membrane potential
Na_2_S	sodium sulfide
NO	nitric oxide
NP	natriuretic peptides
PDE	phosphodiesteras’s
pGC	particulate guanylyl cyclase
PKA	protein kinase A
PKC	protein kinase C
PKG	protein kinase G
sGC	soluble guanylyl cyclase
SK_Ca_	small-conductance Ca^2+^-activated K^+^ channels
SM	smooth muscle
SMC	smooth muscle cells
STOC	spontaneous transient outward currents
SUR	sulfonylurea receptor
TASK	acid-sensitive rectifiers K^+^ channels
TEA	tetraethylammonium
TWIK	lipid sensitive mechano-gated K^+^ channels
TREK	weak inward rectifiers K^+^ channels
UC	umbilical cord
VGCC	voltage-gated Ca^2+^-channels
Zn^+2^	zinc ion
